# Investigation of Erosion/Corrosion Behavior of GRP under Harsh Operating Conditions

**DOI:** 10.3390/polym14245388

**Published:** 2022-12-09

**Authors:** Mohamed K. Hassan, Ahmad Muhammad N. Redhwi, Ahmed F. Mohamed, Ahmed H. Backar, Mohammed Y. Abdellah

**Affiliations:** 1 Mechanical Engineering Department, College of Engineering and Islamic Architecture, Umm Al-Qura University, Makkah 21955, Saudi Arabia; 2 Mechanical Engineering Department, Faculty of Engineering, Sohag University, Sohag 82524, Egypt; 3 Production Engineering Department, Faculty of Engineering, Alexandria University, Alexandria 21544, Egypt; 4 Mechanical Engineering Department, Faculty of Engineering, South Valley University, Qena 83523, Egypt

**Keywords:** GRP, erosion, corrosion, petroleum pipes, harsh environment

## Abstract

Glass-fiber-reinforced pipe (GRP) is a strong alternative to many other materials, such as cast iron and concrete. It is characterized by high corrosion resistance, resulting in good erosion/corrosion. For the erosion/corrosion test, commercially available GRPs were used, which are frequently utilized for oil field wastewater in harsh environments. This type of GRP material was subjected to simulated conditions replicating in situ or harsh environments. An extensive experiment was conducted. Three quantities of abrasive sand (250 g, 400 g and 500 g with a size of 65 µm) were mixed with 0.015 m^3^ of water. The abrasive sand samples were taken at a 90 degree angle from the wall of the cylinder tubes. Three flow rate conditions were selected, 0.01 m^3^/min, 0.0067 m^3^/min and 0.01 m^3^/min, with 10 wt.% chlorine. Furthermore, these tests were conducted at five different times: 1 h, 2 h, 3 h, 4 h and 5 h. The results show that the erosion rate increased both with an increasing amount of abrasive sand and with increasing flow rate. The maximum value for the erosion rate was more than three for a flow rate of 0.015 m^3^ with chlorine for 500 g of sand. The corrosion rate also showed the same trend, with the maximum corrosion rate being reached under the same conditions. It was found that the corrosion rate largely depends on the amount of weight loss, which is an indicator of the erosion effect. Therefore, GFRP provides better erosion/corrosion resistance in a harsh environment or in situ conditions.

## 1. Introduction

Due to their light weight, special modulus of elasticity and high specific impact strength, polymer-based composites are well suited for a variety of industrial and aerospace applications [1,2,3]. The stress corrosion and failure of these reinforced polymers under various environmental conditions have been studied [4,5,6,7]. A thermosetting resin and short or long glass fibers are combined to produce glass-fiber-reinforced plastic, a structural material. When quartz particles are combined with epoxy resin to form the composite matrix, they are sometimes used as reinforcement. Glass-fiber-reinforced pipes are made from this matrix and several laminates of glass-fiber-reinforced polymers. A cheaper alternative to metal pipes is plastic composite pipes. In situations where corrosion, weight and environmental influences play a role, metal pipes are not permitted. There are numerous uses for glass-fiber-reinforced pipes, e.g., for pressure lines and water transmission above and below ground [8,9,10,11].

Nishizaki and Meiarashi [12] investigated the effect of water on durability in a related study. It was found that the flexural strength of GFRP is lower when immersed in water than when merely exposed to ambient moisture. Under different conditions, Bergman and Gunnar [13] were able to control the deterioration of plastics with chlorine, sodium chlorate and chlorine. To determine the general use of plastics in the manufacturing industry and particular settings for plastic applications, the differences between metal and plastic corrosion were analyzed. Geuchy and Hoa [14] conducted an experimental study on the flexural stiffness of thick-walled composite pipes, revealing the relationship between actual and theoretical findings as well as the stark contrast between the concepts of elasticity and material strength. Similarly, Hassan et al. investigated the effect of preconditioning parameters on the ring stiffness properties and fracture toughness of a GFRP material and also derived a numerical FEM to model the ring stiffness [15]. On the other hand, before conducting experiments in water at room temperature, Farshad and Necola [16] conducted compression tests on fiberglass pipes and conditioned the pipe components. The deflection of the specimens was tracked versus time during the test. The results showed that the strength of these materials was reduced due to the environment, including the material and method used. Moreover, three forms of corrosion were distinguished by Hojo et al. [17]: surface reaction, formation of a corrosive layer and corrosion induced by penetration. The long-term stress corrosion of unsaturated glass-fiber-reinforced pipe (GRP) polyester rings was investigated by Farshad and Nicola [18]. These samples were treated with 5% sulfuric acid in the lowest part of the ring. The deformability of the tested part in an acidic environment decreased by about 75% compared to the non-acidic part of the same sample. It was shown that specimen density and dimensional accuracy were directly correlated with orientation [19]. In addition, an angular orientation significantly changed the fracture toughness of specimens with a single-edge notch. The effects of aging and curing in salt water were investigated on polymer composite cylinders used as maritime structures. In terms of mechanical properties, fully cured composite cylinders performed better than partially cured composite cylinders. In addition, salt water aging increased ring strength and stiffness, while it did not affect radial strength. The density and hardness of the identical cylinders were also studied in the presence of salt water and were found to change [19,20]. The effects of salt water on steel pipes coated with fiberglass and epoxy coatings were also studied [21]. Thicker composite repair is required for complete rehabilitation, as demonstrated by the fact that the ring stiffness of the pipes to be rehabilitated increased before and after immersion. The relationship between the ultimate load capacity (UBC) of glass-fiber-reinforced pipe (GFRP) grout and the structural properties of the envelope was simulated [22]. Both the ratio of the volume of the spirally wound layer and the fiber size grew in an expanding pipe, but the UBC increased along with the number of layers in a tube. After immersion in a corrosive medium (sodium chloride solution in water), the fracture strength and mechanical properties of FRP pipes were found to deteriorate [23,24]. Corrosion caused by a corrosive medium was held responsible for this deterioration. On the other hand, the effect of thermal aging on the compression behavior of composites made of interconnected polymer networks reinforced with glass fiber was investigated. The strength of these pipes decreased slightly with increasing temperature and progressive aging.

The behavior of CFRP under solid particle erosion with regard to various laminate orientations and impinging angles was studied by Amkee Kim and Ilhyun Kim [25]. The researchers employed a particle velocity of 70 m/s throughout the experiment for this investigation. The particles employed had a mean diameter of 80 μm, were made of SiC and had orientation angles of 0, 30, 45, 60 and 90 degrees. They concluded that the maximum erosive wear occurred at an impinging angle of 30° and that for unidirectional composites erosion decreased with a decreasing orientation angle from 90° to 45° to 0° while remaining nearly the same for multidirectional composites of [0/90], [45/45], [90/30/30] and [0/60/60]. Thus, erosion is not affected by the orientation of the fiber in multidirectional laminate composites. Additionally, the behavior of solid particle erosion and the mechanism of wear of epoxy-based unidirectional glass-fiber-reinforced plastics (GFRPs) were researched by Y. Fouad et al. [26] The impinging particles utilized by the researchers were silica sand with an average diameter of 150 μm. At various application times and pressures, they used three distinct impinging angle values (30°, 60° and 90°) throughout the experiment. They found that, independent of application duration or pressure levels, the highest degradation occurred at an impinging angle of 60°. They suggested that the material became brittle at this angle, changing its behavior from ductile. In order to increase the wear resistance of the composite, Biswajyoti Pani et al. [27] investigated the effects of adding iron mud mixed with woven glass fiber to an epoxy matrix. They concluded that the inclusion of the reinforcements increased the wear resistance. Another study was conducted on a composite made of wear-resistant polymer. The composite composition, erodent velocity, erodent discharge rate and impinging angle were used as control parameters, and the significance of these components was investigated using ANOVA. Each factor’s three values were utilized. They concluded that the ideal combination of 20% iron mud content, 70 m/s erodent velocity, 12 g/min erodent discharge rate and 90° impinging angle resulted in the minimal erosion of 1.5049 mm^3^/kg.

The mechanical characteristics of the composite cylinder material were assessed using a conventional tensile strength measurement [28], and the static performance of the composite cylinder was calculated using finite element modeling. Through numerical examination, the stress analysis and von Mises stress were achieved. To determine hoop and radial stress caused by the cylinder wall thickness, composite cylinder pipes reinforced with E-glass and T300/934 were studied under pressure [29]. Applications for compressed hydrogen storage employed a composite cylinder with special characteristics [30]. It was thought that the notch effect on the cylinder under internal pressure would emphasize fracture mechanics [31]. The failure mechanisms of the composite cylinder under the influence of low-velocity impact as well as internal pressure were examined using layer-wise theory and a progressive damage model [32]. Composite pipes displayed delamination and severe degradation when subjected to static and impact stresses [33]. A fiber-reinforced composite tube’s critical load for drop weight impact showed rapid destruction and increased incident energy [34]. A glass/carbon functionally graded filament woven composite pipe was studied by Gemi et al. under low-velocity impact and internal pressure [35]. Surface delamination and matrix cracking were seen on the pipes. To forecast the mechanical characteristics and behaviors of the pressurized composite cylinder, useful finite element analysis was carried out. High-density polyethylene (HDPE) was shown to have excellent bearing and high-stress capacities, thus demonstrating significant potential. FEM was suggested as a useful method for pressurized composite cylinder simulation as well [36]. Under external pressure, carbon-reinforced epoxy composite pipes were explored computationally and experimentally, and a finite element model was used to create an assessment damage model. Delamination as well as buckling and in-plane shear modes were seen [37]. Moreover, based on the composite layup of composite pipes, a progressive damage model [38] was created to forecast the failure behavior of composite laminates with various layups and stacking sequences. An effective method for determining the failure processes of carbon-fiber-reinforced pipes was developed using progressive continuum mechanics and experimental findings. In another study by Abdellah et al. [39], analytical and numerical FE models were developed to predict the ring stress and stress intensity factors in cylindrical tubes made with GFRP.

In a previous paper, it was found that there are few studies that deal in depth with the effects of erosion/corrosion behavior of glass-fiber-reinforced FRP pipes. Moreover, the effects of the amount of abrasive agent and the fluid environment on the erosion/corrosion of polymeric material require further investigation; therefore, this study had two main objectives: (1) to experimentally measure weight loss due to the erosive abrasive effect of water with different concentrations and (2) to calculate the erosion and corrosion rates and how they are affected by the three different types of flow rate.

The paper is structured as follows: The first section outlines the erosion/corrosion principles, the second section explains the experimental test ring, the third section presents the method for correlating and extracting the erosion and corrosion rate, and finally, the Results and Discussion are presented.

## 2. Materials and Methods

### 2.1. Glass-Fiber-Reinforced Pipe (GRP)

**Figure 1:**
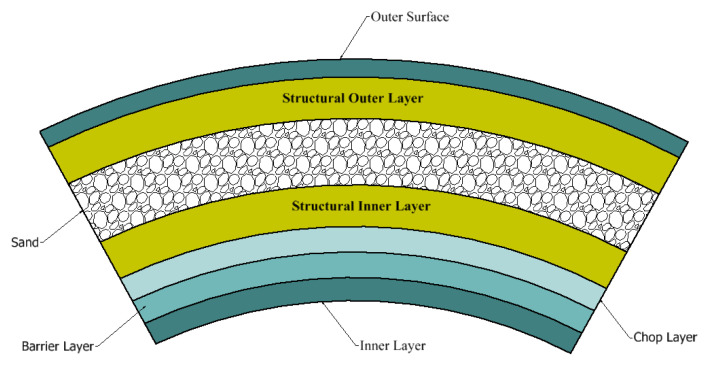
Cross-sectional view of GRPs.

In the current study, glass-fiber-reinforced polymer pipes with a heterogeneous structure of random mats, roving, unsaturated polyester resin and sand were used according to the values shown in Figure 1. Additional details are provided in [23,39,40]. Unsaturated polyester resin binds the fibers in the pipe structure and is resistant to chemicals and the environment. Unsaturated polyester costs less than other resins and is used to make GRP, although it only slightly improves its strength and chemical resistance. It is usually a more affordable choice for low-pressure applications with lower requirements. The common filament winding process was used to manufacture the composite glass fiber pipes. The structure of GRP is complicated, with both inner and outer surface layers. A detailed explanation of the manufacturing process can be found in [21]. The barrier and chaff layers are followed by structural layers on the outer and inner surfaces, between which quartz sand is inserted. The quality of the manufacturing process, the layer thickness, the fiber pretension and the fiber shape are the most important factors that determine the characteristics of the composite material. Using the ASTM D3171-99 standard [41] and ignition removal, these component compositions were produced. In the petroleum industry, these pipes are used in chemical waste pipelines. The elastic properties of the composite glass fiber pipes (GRP) are provided in [23,39,40]. The pipes used for this study were in service for a period of time and then removed from their original location. The overall density of GRP was measured with the roll of the compound as 2.15 g/cm^3^.

### 2.2. Erosion and Corrosion Test

**Figure 2:**
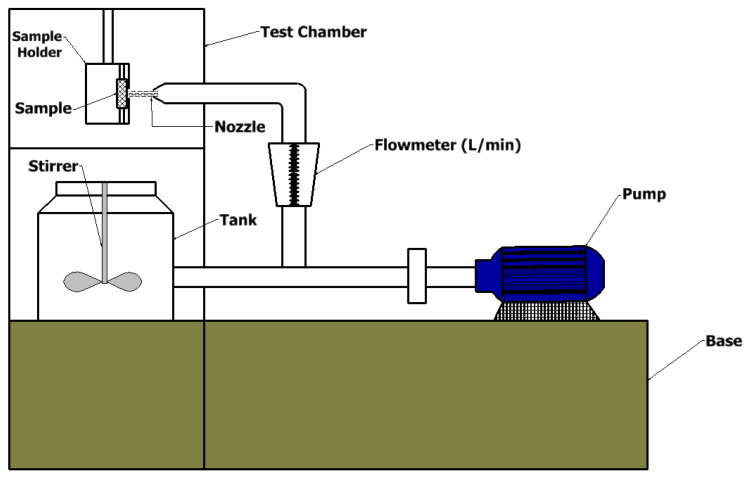
Erosion and correction device.

An erosion/corrosion device was constructed, as shown in Figure 2. It consists of two steel structures installed on four columns welded at each corner. Inside are the pump, the switches, the agitator and the test chamber. At the top is the test chamber in the form of a cubic box measuring 400 × 400 × 400 mm^3^, in which the sample was held on a three-jaw chuck and exposed to the water flow. The walls were attached to the steel beams and columns with super glue, to which silicone was then applied to prevent cracks in the glue. The walls are made of acrylic glass (methyl methacrylate) on four sides to ensure good transparency in observing the experiments. The unit has a cylindrical basin in which water and sand are mixed. In the middle of the basin is an electric mixer that mixes water and sand together. The water–sand mixture flows through the pipe directly to the pump. The electric water pump increases the flow rate of the mixture and sends it to the flow meter and then to the nozzle. The flow meter measures the linear and non-linear volumetric flow. The nozzle directs the water and sand mixture into a linear stream. The water hits the sample at a point and loses its velocity and energy before falling into the basin. This circuit is a closed system. The samples were cut from the wall of a cylinder using a diamond cutter to avoid delamination and damage to the samples, as recommended in papers [15,23,39]. The samples were rectangular in shape with average dimensions of 17 × 14 mm^2^. Three weights of sand were used: 250 g, 400 g and 500 g. These weights were mixed with a constant 0.015 m^3^ of water. The test was conducted at a constant impact angle of 90 degrees, as this angle gives an acceptable performance. The test was conducted for one, two, three, four and five hours, with time being the main variable in the erosion process, as the duration of erosion depends on the time the abrasive hits the sample. The second main variable in the erosion process is the flow rate, so the samples were exposed to two flow rates: 0.0067 m^3^/min and 0.01 m^3^/min. Two corrosive agents were used; at each sand weight, chlorine was used at a concentration of 10 wt.% and a flow rate of 0.01 m^3^/min. The average size of the sand particles was 65 µm. As reported by Abdellah et al. [23], chlorine is the main factor responsible for the corrosion of this material, especially in the harsh environment of underground oil fields.

Weight loss can be calculated using the cumulative value of weight loss in each hour and subsequent hours, as follows (ASTM G31-72 standard) [42].

(1) $$W_{loss\left(i\right)}=\underset{i,j}{\sum }(w_{i-1}+w_{j})$$

where *i* = 1, 2 to 5 and *j* = 1, 2, 3 to 5 are the two adjacent hours.

Due to variations in the velocities of a corrosive fluid and a metal surface, erosion corrosion refers to the acceleration or increase in the intensity of an attack on a metal. This velocity is often high, and mechanical wear and abrasion effects often occur. When the flow pattern is interrupted, such as in a pipe downstream of a constriction, obstructed or bent, this causes severe turbulence, leading to erosion corrosion [43], as dissolved ions or dimensionally stable corrosion products physically erode from the metal surface. In the case of soft metals or when a protective layer on the metal surface is destroyed, erosion corrosion easily occurs. Increased general corrosion is usually the result of erosion corrosion. In the process of erosion and corrosion, solid particles or air bubbles in liquids can cause great damage. Generally, erosion corrosion can be recognized by a characteristic flow pattern, e.g., smooth pits, grooves, waves or gullies [43].

The erosion rate *E* can be measured according to Equation (1) [43], as follows:

(2) $$E=\frac{weight\;loss\;due\;to\;errosion}{time\;taken\;to\;errode}=\frac{W_{loss}}{T}$$

The area exposed to erosion *At* for the material can be calculated using Equation (2) [42], as follows:

(3) $$At=\frac{\pi }{\sin\alpha }$$

where α is the angle of impact. Moreover, the relationship between weight loss and corrosion can be obtained using Equation (3) [42], as follows:

(4) $$\mathrm{Corrosion}\;\mathrm{rate}=Cr=\frac{weight\;loss\;due\;to\;errosion}{time\;taken\;to\;errode}=\frac{K\times w}{D\times At\times T}$$

where *K* is constant (876 × 10^4^), *w* is weight loss, *D* is material density (GRP density is listed in Table 1), and *T* is time exposure (hours). The maximum exposed area according to Equation (2) was (785 × 10^−3^) m^2^.

## 3. Results and Discussion

Table 1, Table 2 and Table 3 show the data for the erosion/corrosion test. It was found that the maximum value of erosion wear was 1.15% for the sample eroded with 250 g of sand at 0.01 m^3^/min, while the minimum value was about 0.05% for the sample eroded with 250 g of sand at 0.0067 m^3^/min. This was because corrosion occurred more easily for samples with a lower strength reinforcing phase. There was a negative sign in the case of erosion conditions at 0.01 m^3^/min + 10 wt.%. This is because chlorine greatly affects the bonding of the silica sand particles used in the manufacture of GRPs. The polymer resin reacts chemically with the chlorine, leaving gaps between the sand particles that act like voids into which water penetrates; hence, their weight increases after the test at shallow depths of the ablated surfaces, as noted by Abdellah et al. [23,44]. On the other hand, this phenomenon disappeared when greater amounts of eroded sand were used, including 400 g or even 500 g, because the high erosion rate in these conditions did not give the material sufficient time to trap water inside. The wear rate due to erosion depends on the kinetic energy of particles (impact velocity), impact angle and abrasive agent size. The erosion corrosion mechanism occurs through the GRP material. The passive thin layer on the surface of the GRP pipe is a non-active chemically protective barrier layer between the corrosive water and GRP substrate with low mechanical strength; therefore, it can be broken off by the high impact or strike velocity of abrasive sand, after which the GRP beneath forms another passive layer [45].

**Figure 3:**
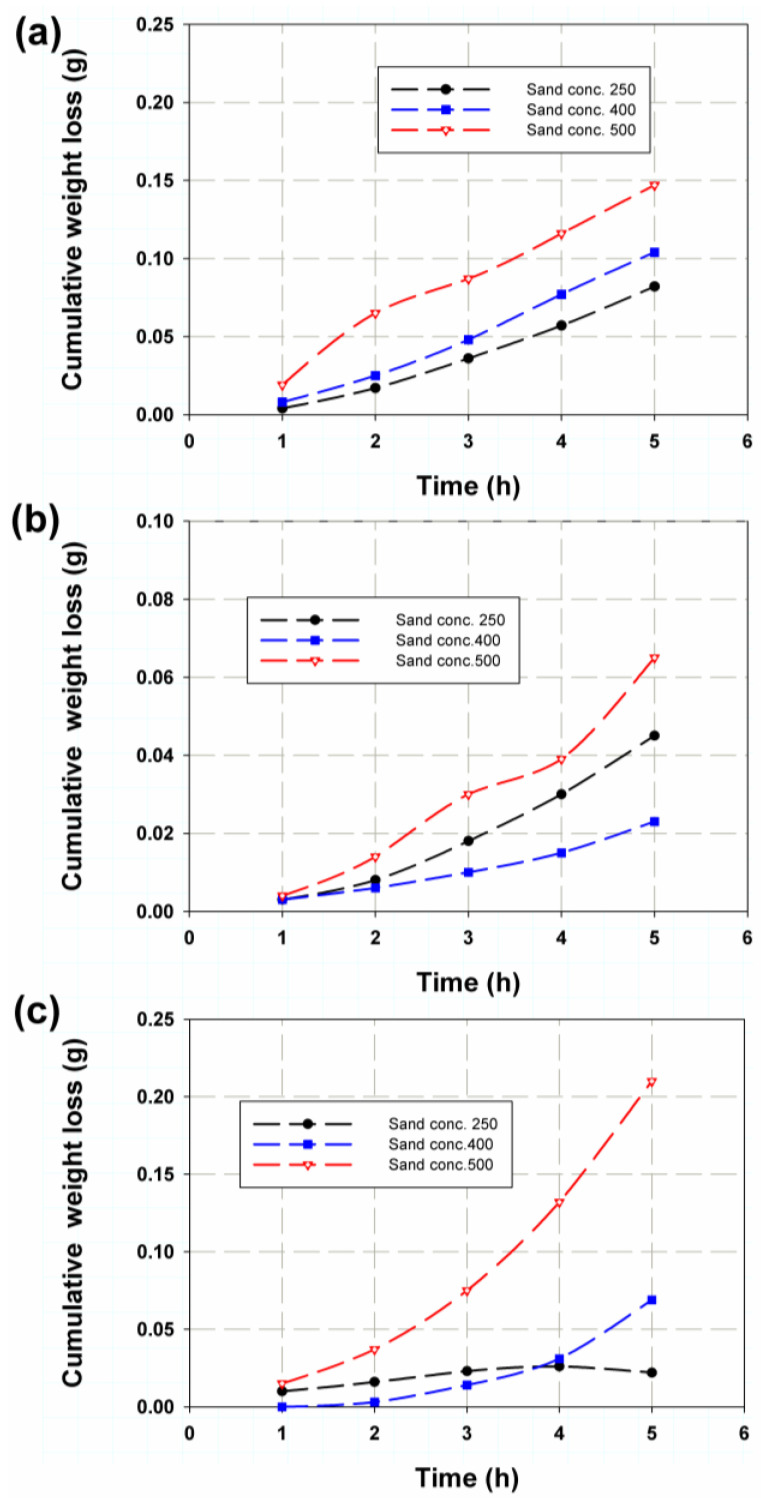
Cumulative weight loss versus time for (**a**) 0.01 m^3^/min, (**b**) 0.0067 m^3^/min and (**c**) 0.01 m^3^/min and 10 wt.% Cl flow rates.

The trend of erosion performance of the permeable table for each sample was unclear for the effect of flow rate or sand particle quantity. Figure 3 shows the cumulative weight loss based on Equation (1). It was found that at a flow rate of 0.01 m^3^/min, as shown in Figure 3a, the sand quantity increased almost linearly with hours. A maximum of 0.15 g was reached for 500 g of sand (red line), a maximum of 0.104 g for 400 g and a maximum of 0.082 g for 250 g. This is a logical trend, as a larger amount of abrasive sand can result in greater depth. However, the increase in depth was less than the increase in the amount of abrasive sand. This was due to cracks in the GRP sample caused by the deformation of the material. Therefore, any increase in the number of particles after reaching the deformation limit would result in a limited increase in erosion depth [46]. However, there was a deviation from this trend at 0.0067 m^3^/min and 0.01 m^3^/min with 10 wt.% Cl (see Figure 3b,c), where the 250 g of sand gave a mean value between 500 g and 400 g of sand. As shown in Figure 3c, the cumulative weight loss for 250 g of sand decreased after 4 h, which could have been due to the fact that after 4 h, the erosion rate and thus the corresponding erosion depth reached the glass fiber layers.

**Figure 4:**
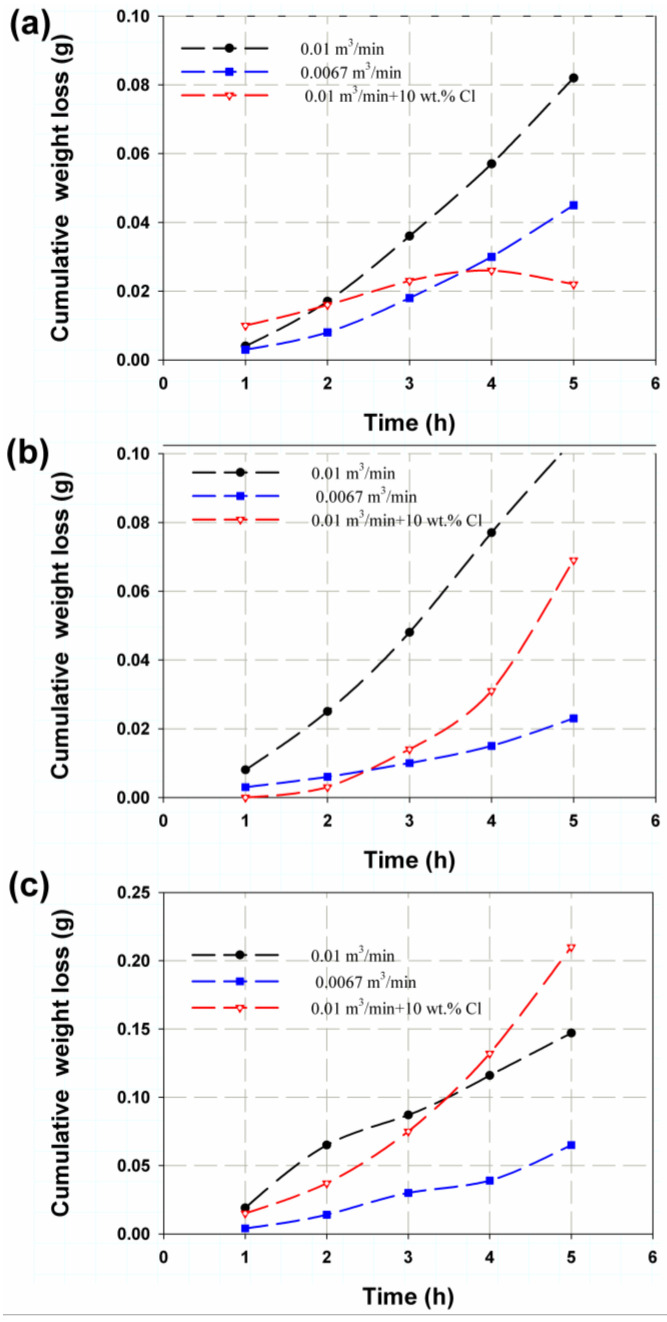
Cumulative weight loss versus time for (**a**) 250 g, (**b**) 400 g and (**c**) 500 g of sand mass.

The effect of flow rate has a great influence on cumulative weight loss. As can be seen in Figure 4, the weight loss increased with increasing flow velocity due to the increase in impact velocity. This can be attributed to the fact that high velocity with abrasive sand generates huge momentum with high kinetic energy, impacting the sample surfaces and then eroding the weakened layers [47], so as the amount of sand increases, the number of impact points increases, as does the total mass loss. At the flow rate AA (0.01 m^3^/min and 10 wt.% Cl), however, a different trend was seen. In the first hour, the weight loss was highest for the flow rate AA, and in the second hour, it was very close to 0.01 m^3^/min (see Figure 4a), while it reached its lowest value after 4 h. The weight loss at the flow rate AA for 400 g of sand, as shown in Figure 4b, was lowest in the first two hours and increased sharply from 0.0067 m^3^/min to 0.01 m^3^/min. This may be due to the fact that chlorine increases corrosion, which plays a significant role in enlarging cavities in the nearest resin layers, as these are then penetrated by water. These voids fluctuated during the test and were not constant but depended on how and when the reaction took place. In addition, some of these near-surface voids are removed over time, which affects the weight loss. Those corrosion products subjected to high flow velocities or drop impact are quickly removed by shear stress [48].

**Figure 5:**
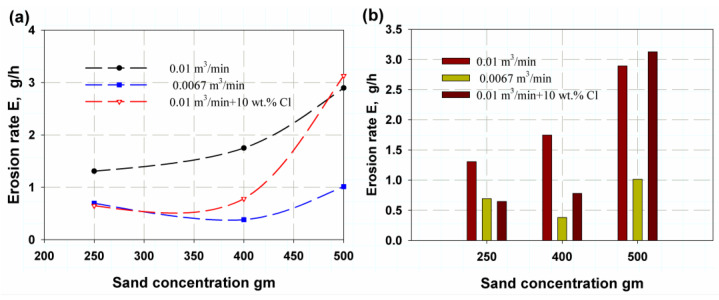
Erosion rate variation with amount of abrasive sand: (**a**) total trend, (**b**) maximum value.

**Figure 6:**
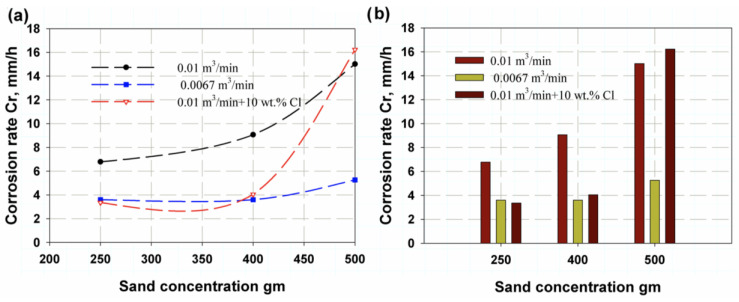
Corrosion rate variation with abrasive sand amount: (**a**) total trend, (**b**) maximum value.

**Figure 7:**
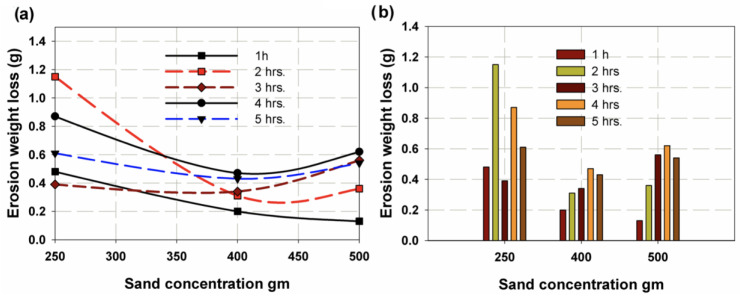
Erosion weight loss variation with time of impact (**a**) total trend, (**b**) maximum value.

Figure 5 shows the erosion rate as a function of sand concentration or amount. It was found that as the flow rate increased, permeable sand obtained the same results for erosion rate (see Figure 5), with maximum erosion rates of 6.78 g/h, 9.06 g/h and 15.01 g/h for 250 g, 400 g and 500 g of sand, respectively, at a flow rate of 0.01 m^3^/min. On the other hand, the minimum values for a flow rate of 0.0067 m^3^/min were 3.59 g/h, 3.59 g/h and 5.25 g/h for 250 g, 400 g and 500 of g sand, respectively. The mean values obtained for the flow rate AA were 3.35 g/h, 4.04 g/h and 16.22 g/h for 250 g, 400 g and 500 g of sand, respectively. The deviation for the flow rate AA was attributed to the corrosion effect of the chlorine. These results are plotted in Figure 6a, where it can be seen that the corrosion rate showed the same trend as the erosion effect, but with different values. In Figure 6b, it is clear that as the amount of sand increased and the flow rate increased from 0.01 m^3^/min with or without chlorine, so too did the corrosion rate. The effect of impact time is illustrated in Figure 7a, where it can be seen that it reached a maximum value for 250 g of sand and then largely decreased for 400 g and continued to decrease for 500 g for all impact times except at 4 h, where it continued decrease. This is because 250 g of sand leads to larger inter-distance between sand particles, making the roll like coarse emery paper, whereas for an increasing amount of abrasive sand, the distance between particles is smaller, making rolls more similar to soft emery paper. Figure 7b shows that for 250 g of abrasive sand, the erosion weight loss has the following sequence: 2 h, 4 h, 5 h, 3 h and 1 h. Meanwhile, at 400 g and 500 g of abrasive sand, erosion loss increases with increasing time.

**Figure 8:**
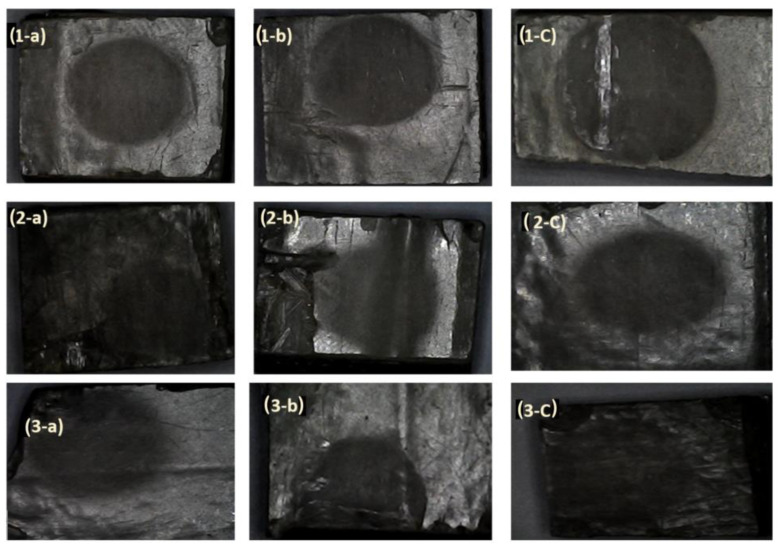
Failure mode at 2 h for (**a**) 250 g, (**b**) 400 g and (**c**) 500 g of sand at (**1**) 0.01 m^3^/min, (**2**) 0.0067 m^3^/min and (**3**) 0.01 m^3^/min +10 wt.% Cl.

**Figure 9:**
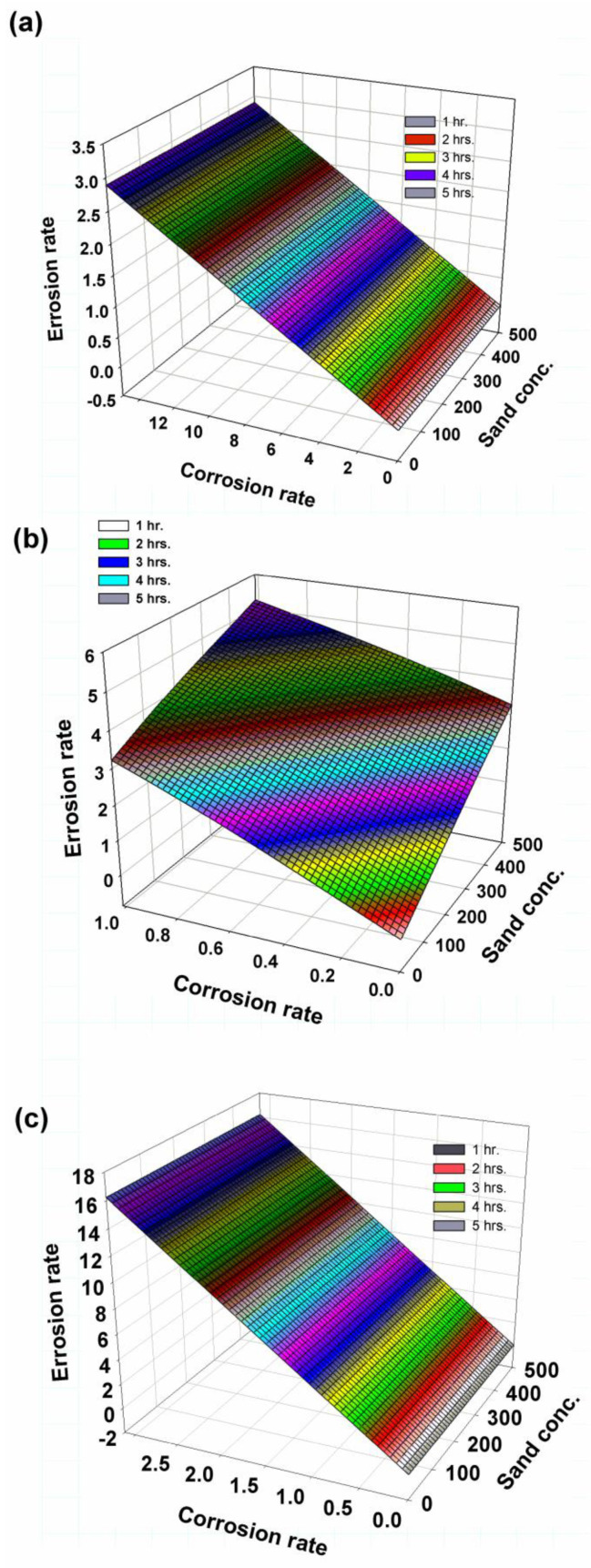
Three-dimensional diagram of sand concentration with erosion and corrosion rates: (**a**) 0.01 m^3^/min, (**b**) 0.0067 m^3^/min, (**c**) 0.01 m^3^/min +10 wt.% Cl.

The failure modes for 2 h of corrosion for 250 g, 400 g and 500 g of sand are shown in Figure 8(1-a–1-c). It was observed that the impressions in the impact area were darker and almost circular. Moreover, the damaged areas at the ends of the specimens at a flow rate of 0.0067 m^3^/min can be observed in Figure 8(2-a–2-c); the damage was severe at the flow rate AA, as seen in Figure 8(3-a–3-c). The relationship between sand concentration, erosion rate and corrosion rate is shown in Figure 9. The failure modes, due to erosion corrosion, are like fingerprints in most specimens. On the other hand, for GRP, which is considered a linear material or brittle material, the failure occurred due to the impact of abrasive particles, which resulted in surface cracking and frittering into micro-sized GRP pieces [49]; therefore, kinetic energy is the main erosion factor, for which the maximum loss of weight occurred at the impact angle = 90°, as cited by Hamed and Tabakoff [50]. An increasing amount of corrosion was found to coalesce around and within the impact area. In this case, many particles of abrasive sand on the GRP surface produced a repeated plastic deformation; this in turn led to fatigue cracking and hence crack coalescence, and the fatigue cracks spread and interlaced with each other over time. Therefore, the surface was worn and torn [51].

## 4. Conclusions

GRPs are nowadays a strong and competitive alternative to monotonous metals, especially steel, due to their high resistance to corrosion and erosion. The erosion corrosion behavior of GRP material installed in a harsh environment was studied and fully qualified. The erosion rate was highly dependent on the flow rate of the erosive fluid and the sand concentration. At a 0.01 m^3^/min flow rate, the highest value of corrosion and erosion was seen regardless of the amount and concentration of abrasive sand. It was found that the corrosion factor had a maximum value of 16.08 mm/h and a minimum value of 3.11 mm/h; this corresponded to the maximum erosion rate of 3.11 g/h and minimum erosion rate of 0.063 g/h for 500 g and 250 g of sand, respectively. There was a robust relationship between the corrosion and erosion of the FRP material and the amount of abrasive sand used. In addition, chlorine was found to have a major effect on the corrosion and erosion behavior of the material; a small amount of chlorine, 10 wt.%, gave the highest corrosion and erosion rate with 500 g of abrasive sand. It was suggested that the amount of resin on the surface of the material should be increased to protect the inner material, which contains quartz, from chlorine, especially in a harsh environment where fresh water is exposed to chlorine gas. It was finally reported that most GRP failures are concerned with corrosion/erosion deterioration, which degrades the material strength. Moreover, we suggest future work studies the effect of the impact angle, abrasive sand size and impact velocity on the corrosion/erosion performance of GRPs.

## Figures and Tables

**Table 1:** Test data at a flow rate of 0.01 m^3^/min.

Sand Conc.	Time (H)	Weight Before	Weight After	Weight Difference (g)	Weight Difference %
250	1	4.00	3.98	0.02	0.48
250	2	4.00	3.95	0.05	1.15
250	3	5.62	5.60	0.02	0.39
250	4	3.35	3.32	0.03	0.87
250	5	5.11	5.08	0.03	0.61
400	1	4.00	3.99	0.01	0.20
400	2	5.54	5.52	0.02	0.31
400	3	6.82	6.80	0.02	0.34
400	4	6.17	6.14	0.03	0.47
400	5	6.26	6.23	0.03	0.43
500	1	3.12	3.12	0.00	0.13
500	2	3.61	3.59	0.01	0.36
500	3	3.38	3.36	0.02	0.56
500	4	3.41	3.39	0.02	0.62
500	5	4.61	4.59	0.03	0.54

**Table 2:** Test data at a flow rate of 0.0067 m^3^/min.

Sand Conc.	Time (H)	Weight Before	Weight After	Weight Difference (g)	Weight Difference %
250	1	5.621	5.618	0.003	0.053
250	2	4.704	4.699	0.005	0.106
250	3	3.003	2.993	0.010	0.333
250	4	3.705	3.693	0.012	0.324
250	5	3.220	3.205	0.015	0.466
400	1	3.230	3.227	0.003	0.093
400	2	3.223	3.220	0.003	0.093
400	3	4.491	4.487	0.004	0.089
400	4	5.192	5.187	0.005	0.096
400	5	6.851	6.843	0.008	0.117
500	1	4.590	4.586	0.004	0.087
500	2	6.941	6.931	0.010	0.144
500	3	3.054	3.038	0.016	0.524
500	4	4.347	4.338	0.009	0.207
500	5	4.026	4.000	0.026	0.646

**Table 3:** Test data at a flow rate of 0.01 m^3^/min + (10 wt.% Cl).

Sand Conc.	Time (H)	Weight Before	Weight After	Weight Difference (g)	Weight Difference %
250	1	3.558	3.568	−0.010	−0.281
250	2	5.922	5.928	−0.006	−0.101
250	3	4.311	4.318	−0.007	−0.162
250	4	4.615	4.618	−0.003	−0.065
250	5	5.575	5.571	0.004	0.072
400	1	4.854	4.854	0.000	0.000
400	2	4.557	4.554	0.003	0.066
400	3	4.661	4.650	0.011	0.236
400	4	4.864	4.847	0.017	0.350
400	5	5.621	5.583	0.038	0.676
500	1	4.638	4.623	0.015	0.323
500	2	3.602	3.580	0.022	0.611
500	3	6.170	6.132	0.038	0.616
500	4	6.247	6.190	0.057	0.912
500	5	5.870	5.792	0.078	1.329

## Data Availability

The data presented in this study are available on request from the corresponding author.

## References

[1] Fukushima K., Cai H., Nakada M., Miyano Y. Determination of time-temperature shift factor for long-term life prediction of polymer composites. Proceedings of the ICCM-17-17th International Conference on Composite Materials.

[2] Plota A., Masek A. (2020). Lifetime Prediction Methods for Degradable Polymeric Materials—A Short Review. Materials.

[3] Abdellah M.Y. (2018). Comparative study on prediction of fracture toughness of CFRP laminates from size effect law of open hole specimen using cohesive zone model. Eng. Fract. Mech..

[4] Julius M.J. (2003). Time, Temperature and Frequency Viscoelastic Behavior of Commercial Polymers.

[5] Wang J., Parvatareddy H., Chang T., Iyengar N., Dillard D., Reifsnider K. (1995). Physical aging behavior of high-performance composites. Compos. Sci. Technol..

[6] Yao J., Ziegmann G. (2006). Equivalence of moisture and temperature in accelerated test method and its application in prediction of long-term properties of glass-fiber reinforced epoxy pipe specimen. Polym. Test..

[7] Barbero E.J., Julius M.J. (2004). Time-temperature-age viscoelastic behavior of commercial polymer blends and felt filled polymers. Mech. Adv. Mater. Struct..

[8] Feng C.-W., Keong C.-W., Hsueh Y.-P., Wang Y.-Y., Sue H. (2005). Modeling of long-term creep behavior of structural epoxy adhesives. Int. J. Adhes. Adhes..

[9] Chen M. (1991). Accelerated Viscoelastic Characterization of E-glass/Epoxy Composite.

[10] Goertzen W., Kessler M. (2006). Creep behavior of carbon fiber/epoxy matrix composites. Mater. Sci. Eng. A.

[11] Miyano Y., Nakada M., Sekine N. (2005). Accelerated testing for long-term durability of FRP laminates for marine use. J. Compos. Mater..

[12] Nishizaki I., Meiarashi S. (2002). Long-term deterioration of GFRP in water and moist environment. J. Compos. Constr..

[13] Bergman G. Managing corrosion on plastics-an analysis of experience from industrial applications. Proceedings of the 55th NACE International Annual Corrosion Conference and Exposition.

[14] Ahmad M.G., Hoa S. (2016). Flexural stiffness of thick walled composite tubes. Compos. Struct..

[15] Hassan M.K., Mohamed A.F., Khalil K.A., Abdellah M.Y. (2021). Numerical and Experimental Evaluation of Mechanical and Ring Stiffness Properties of Preconditioning Underground Glass Fiber Composite Pipes. J. Compos. Sci..

[16] Farshad M., Necola A. (2004). Effect of aqueous environment on the long-term behavior of glass fiber-reinforced plastic pipes. Polym. Test..

[17] Hojo H., Tsuda K., Kubouchi M., Kim D.S. (1998). Corrosion of plastics and composites in chemical environments. Met. Mater..

[18] Farshad M., Necola A. (2004). Strain corrosion of glass fibre-reinforced plastics pipes. Polym. Test..

[19] Stoia D.I., Marsavina L., Linul E. (2020). Mode I Fracture Toughness of Polyamide and Alumide Samples obtained by Selective Laser Sintering Additive Process. Polymers.

[20] GÜNÖZ A., Kepir Y., Memduh K. (2022). The investigation of hardness and density properties of GFRP composite pipes under seawater conditions. Turk. J. Eng..

[21] Shi H., An Z., Gao R. (2020). Simulation of Mechanical Behavior and Structural Analysis of Glass Fiber Reinforced Mortar Pipes. Rev. Romana De Mater..

[22] Srinivasan T., Suresh G., Ramu P., Vignesh R., Harshan A.V., Vignesh K. (2021). Effect of hygrothermal ageing on the compressive behavior of glass fiber reinforced IPN composite pipes. Mater. Today Proc..

[23] Abdellah M.Y., Hassan M.K., Alsoufi M.S. (2016). Fracture and Mechanical Characteristics Degradation of Glass Fiber Reinforced Petroleum epoxy Pipes. J. Manuf. Sci. Prod..

[24] Seleem A.-E.H.A. (2015). Failure and Corrosion Analysis of Composite Glass Fiber Reinforced Pipe Lines. Ph.D. Thesis.

[25] Kim A., Kim I. (2009). Solid particle erosion of CFRP composite with different laminate orientations. Wear.

[26] Fouad Y., El-Meniawi M., Afifi A. (2011). Erosion behaviour of epoxy based unidirectionl (GFRP) composite materials. Alex. Eng. J..

[27] Pani B., Chandrasekhar P., Singh S. (2018). A study on erosion wear behavior of iron-mud/glass fiber reinforced epoxy composite. IOP Conference Series: Materials Science and Engineering.

[28] Nouri M., Ashenai-Ghasemi F., Rahimi-Sherbaf G., Kashyzadeh K.R. (2020). Experimental and Numerical Study of the Static Performance of a Hoop-Wrapped CNG Composite Cylinder Considering Its Variable Wall Thickness and Polymer Liner. Mech. Compos. Mater..

[29] Sülü İ.Y., Temiz Ş. (2020). Mechanical behavior of pressurized composite pipes made of various materials. Mater. Test..

[30] Sharma P., Bera T., Semwal K., Badhe R.M., Sharma A., Ramakumar S., Neogi S. (2020). Theoretical analysis of design of filament wound type 3 composite cylinder for the storage of compressed hydrogen gas. Int. J. Hydrogen Energy.

[31] Atalay O., Toktas I. (2021). Notch (stress concentration) factor estimation of a cylinder under internal pressure using different approaches. Mater. Test..

[32] Rafiee R., Rashedi H., Rezaee S. (2020). Theoretical study of failure in composite pressure vessels subjected to low-velocity impact and internal pressure. Front. Struct. Civ. Eng..

[33] Alderson K., Evans K. (1992). Failure mechanisms during the transverse loading of filament-wound pipes under static and low velocity impact conditions. Composites.

[34] Guades E., Aravinthan T., Manalo A., Islam M. (2013). Experimental investigation on the behaviour of square FRP composite tubes under repeated axial impact. Composite Structures.

[35] Gemi L., Kara M., Avci A. (2016). Low velocity impact response of prestressed functionally graded hybrid pipes. Compos. Part B Eng..

[36] Velosa J., Nunes J.P., Antunes P., Silva J., Marques A. (2009). Development of a new generation of filament wound composite pressure cylinders. Compos. Sci. Technol..

[37] Almeida J.H.S., Ribeiro M.L., Tita V., Amico S.C. (2016). Damage and failure in carbon/epoxy filament wound composite tubes under external pressure: Experimental and numerical approaches. Mater. Des..

[38] Huang Z., Qian X., Su Z., Pham D.C., Sridhar N. (2020). Experimental investigation and damage simulation of large-scaled filament wound composite pipes. Compos. Part B Eng..

[39] Abdellah M., Alfattani R., Alnaser I., Abdel-Jaber G. (2021). Stress Distribution and Fracture Toughness of Underground Reinforced Plastic Pipe Composite. Polymers.

[40] Faria H.Q.d. (2005). Failure analysis of GRP pipes under compressive ring loads. Master’s Thesis.

[41] (1999). Standard Test Methods for Constituent Content of Composite Materials.

[42] Prasojo B., Kurniyanto H.B., Azis R.T., So’Im S., Haqin A.R. (2019). Effect of Flow Rate and Temperature on Erosion Corrosion Rate of Crude Palm Oil Against Elbow A53 Grade B Carbon Steel Material. IOP Conference Series: Materials Science and Engineering.

[43] Blue M.-L. How to Calculate Erosion Rate. https://sciencing.com/calculate-erosion-rate-6118473.html.

[44] Abdellah M.Y. (2017). Delamination modeling of double cantilever beam of unidirectional composite laminates. J. Fail. Anal. Prev..

[45] Patel C. (2022). https://melezy.com/erosion-corrosion-mechanism-factors-prevention/.

[46] Abouel-Kasem A. (2011). Particle size effects on slurry erosion of 5117 steels. J. Tribol..

[47] Frosell T., Fripp M., Gutmark E. (2015). Investigation of slurry concentration effects on solid particle erosion rate for an impinging jet. Wear.

[48] Ramajo D., Raviculé M., Benini R., Damian S.M., Storti M., Nigro N. (2009). Diagnose and Prediction of Erosion-Corrosion Damage in a Pipeline Transporting Reduced Crude Oil from the Heater to the Vacuum Distillation Tower. Mecánica Comput..

[49] Mazumder Q.H., Shirazi S.A., McLaury B.S., Shadley J.R., Rybicki E.F. (2005). Development and validation of a mechanistic model to predict solid particle erosion in multiphase flow. Wear.

[50] Hazuku T., Takamasa T., Hibiki T., Ishii M. (2007). Interfacial area concentration in annular two-phase flow. Int. J. Heat Mass Transf..

[51] Wen D.-C. (2009). Erosion–corrosion behavior of plastic mold steel in solid/aqueous slurry. J. Mater. Sci..

